# Pioglitazone ameliorates glomerular NLRP3 inflammasome activation in apolipoprotein E knockout mice with diabetes mellitus

**DOI:** 10.1371/journal.pone.0181248

**Published:** 2017-07-14

**Authors:** Yao Wang, Bo Yu, Li Wang, Ming Yang, Zhiyin Xia, Wei Wei, Fengyu Zhang, Xiaochen Yuan

**Affiliations:** 1 Department of nephrology, the affiliated hospital of Yangzhou University (Yangzhou NO.1 people’s hospital), Yangzhou University, Yangzhou, Jiangsu, China; 2 Department of emergency, the affiliated hospital of Yangzhou University (Yangzhou NO.1 people’s hospital), Yangzhou University, Yangzhou, Jiangsu, China; 3 Department of cardiology, the affiliated hospital of Yangzhou University (Yangzhou NO.1 people’s hospital), Yangzhou University, Yangzhou, Jiangsu, China; 4 Central lab, the affiliated hospital of Yangzhou University (Yangzhou NO.1 people’s hospital), Yangzhou University, Yangzhou, Jiangsu, China; University of the Pacific, UNITED STATES

## Abstract

**Objective:**

The NLRP3 inflammasome plays an important role in the pathogenesis of inflammation in diabetic nephropathy (DN). Pioglitazone (PIO) has been found to exert an anti-inflammatory effect in patients with diabetes mellitus, but it is still unclear whether PIO exhibits a similar effect in DN. We aimed to explore the effect and underlying mechanism of PIO on DN, as well as investigate if NLRP3 is a pharmacologic target of PIO.

**Methods:**

We divided 48 apolipoprotein E (apoE) (-/-) mice into 4 groups: apoE (-/-), apoE (-/-) with PIO, diabetic apoE (-/-), and diabetic apoE (-/-) with PIO. Wild type male C57BL/6 mice were used as controls (n = 8 per group). After 8 weeks of PIO treatment, we examined the baseline characteristics and metabolic parameters of each group, and we used enzyme-linked immunosorbent assay (ELISA), western blot, and immunohistochemical staining to evaluate the expression levels of advanced glycation end products (AGEs), receptor for advanced glycation end products (RAGE), NLRP3, nuclear factor—kappa B (NF-*κ*B), caspase-1, interleukin (IL)-18, and IL-1β in each group.

**Results:**

Compared to the diabetic apoE (-/-) group, PIO treatment decreased blood glucose, cholesterol, serum blood urea nitrogen (BUN), and creatinine levels. It also depressed the glomerular mesangial expansion. PIO down-regulated expression of AGEs, RAGE, and NF-*κ*B, all of which further depressed NLRP3, caspase-1, IL-18, and IL-1β levels.

**Conclusion:**

Pioglitazone can ameliorate diabetic renal damage, and this effect is related to the inhibition of renal AGE/RAGE axis activation and the down-regulation of NF-*κ*B expression. These effects lead to a decline in NLRP3 levels and downstream secretion of inflammatory cytokines.

## Introduction

The prevalence of diabetes mellitus has been growing worldwide, but available treatment for this disease remains limited. Despite the decline in mortality from macrovascular diabetic complications, diabetic nephropathy (DN) is still a leading cause of end-stage renal disease (ESRD), and the number is still rising. It has been reported that once nephropathy develops in patients with diabetes, approximately 40% inevitably progress to ESRD [1].

A growing body of evidence suggests that advanced glycation end products (AGEs) and receptor for advanced glycation end products (RAGE) interaction produce oxidative stress, as well as inflammatory and fibrotic reactions, causing progressive disruption of normal renal architecture and the loss of renal function in patients with diabetes. AGEs enhance mitochondrial reactive oxygen species (ROS) through binding to RAGE, and activation of the AGE/RAGE axis in podocytes enhances the expression of NLRP3 and cleaved interleukin (IL)-1β [2]. Uric acid-induced tubular nuclear factor—kappa B (NF-*κ*B) expression also increases NLRP3 activation in macrophages, accompanied by tubulointerstitial fibrosis and macrophage infiltration during the course of DN [3]. These findings indicate that both the AGE/RAGE axis and NF-*κ*B up-regulation can activate the NLRP3 inflammasome during chronic kidney disease. Activation of the NLRP3 inflammasome promotes renal injury through the up-regulation of IL-1β and IL-18 [4], and enhances IL-1β production in diabetes [5]. Upon detection of cellular stress, NLRP3 recruits and binds with apoptosis-associated speck-like protein contains caspase recruitment domain (ASC) and caspase-1, resulting in the activation of caspase-1, the maturation of IL-1β and IL-18, and the recruitment of inflammatory cells [6]. This is evident in diabetic mouse models, in which db/db mice have markedly higher serum levels of IL-1β, IL-18, and renal NLRP3 expression compared to their wild type littermates [7]. Consequently, targeting the inflammasome pathway may be a potential therapeutic approach for DN.

Peroxisome proliferator-activated receptors (PPARs) are nuclear receptors that participate in the transcriptional modulation of diverse cellular functions including lipid metabolism, glucose homeostasis, differentiation, and inflammation [8]. Recent studies have highlighted a favorable effect for PPAR-γ agonists, such as pioglitazone (PIO), in patients with DN and non-diabetic nephropathy [9,10]. In the kidney, PIO significantly reduces macrophage infiltration and activation of NF-*κ*B accompanied by a decrease in expression of type IV collagen, plasminogen activator inhibitor-1 (PAI-1), and tumor growth factor beta-1 (TGFβ-1) [11]. PPAR-γ agonists can also antagonize AGEs in renal tubular cells by suppressing expression of RAGE [12]. However, none of the existing studies addresses whether PIO can down-regulate the NLRP3-inflammasome and suppress the inflammatory status. In this study, we investigated the reno-protective effect of PIO in apolipoprotein E (apoE)-knockout (-/-) mice with diabetes, with a particular emphasis on the relationship between PIO and alterations in NLRP3 inflammasome activation, as well as the underlying mechanisms.

## Materials and methods

### 1. Animals model

The study protocols conformed to the Guide for the National Institutes of Health Guide for the Care and Use of Laboratory Animals (NIH Publications No. 8023, revised 1978). All animal procedures and protocols were approved by the Special Committee on Animal Welfare of Yangzhou University, Jiangsu, China. Animals were treated based on the regulations for the administration of affairs concerning experimental animals. 48 apolipoprotiein E (apoE) (-/-) mice (6 weeks old) were housed in the Animal Center of Yangzhou NO.1 People’s Hospital, according to the policy of the Committee for Animal Usage. ApoE (-/-) mice were fed a high glucose and high fat chow, consisting of 15% sucrose, 4% cholesterol, 10% lard, 0.3% bile salt, 10% egg yolk powder, and 60.7% basic feed. ApoE (-/-) mice were further divided into 4 groups: apoE (-/-) (group A); apoE (-/-) with PIO (group B); diabetic apoE (-/-) (group C); and diabetic apoE (-/-) with PIO (group D). Mice were intraperitoneally administered a single injection of either streptozotocin (80 mg/kg; S0130; Sigma-Aldrich, MO) diluted in 0.1 M citrate buffer at pH 4.5 (diabetic) or citrate buffer only (non-diabetic). Male C57BL/6 mice (6 weeks old) served as a control group (group E), and were fed normal chow. All blood samples were collected from the tail vein. Plasma glucose concentrations were obtained using the glucose oxidase method on a glucose analyzer (Accu-check Advantage; Roche, Mississauga, ON) 3 days after streptozotocin injection, and mice with a glucose level over 16.7 mmol/L were considered diabetic mice and included in the study. Plasma glucose was monitored once every week. Body weight, fluid, and food intake were also recorded. PIO (10 mg/kg/day; 1539905, Sigma-Aldrich, MO) was intraperitoneally injected into all mice in groups B and D every day until sacrifice at 8 weeks, with DMSO used as a control.

### 2. Biochemical indices

The animals were anesthetized with an intraperitoneally injected mixture of ketamine (80 mg/kg) and xylazine (12 mg/kg).

Eyeball exsanguination was performed to obtain blood triglyceride, cholesterol, serum blood urea nitrogen (BUN), and creatinine measurements using automatic analyzers (Hitachi, Tokyo, Japan). Proteins were extracted from the renal cortex from each group (n = 5–6/group). AGEs levels were detected using a mouse enzyme-linked immunosorbent assay (ELISA) Kit (Cat NO CSB-E09414m CUSABIO CN) according to the manufacturer’s protocol.

### 3. Western blot

After myocardial perfusion with phosphate buffered solution, kidneys were harvested, and renal cortical homogenates were pre-processed using a Total Protein Extraction Kit (KeyGEN, Nanjing, China) to detect RAGE and NF-KB levels. Protein concentrations were determined by the Bradford-based method (Bio-rad), and specimens were analyzed using a Pierce BCA Protein Assay Kit (Thermo Scientific, Rockford, USA). Samples were then separated by 12% SDS-PAGE and transferred to nitrocellulose membranes, to reduce background staining, followed by incubation with 5% Bull Serum Albumin in Tris Buffer Solution containing 0.1% Tween 20 for 60 minutes. The membranes were further incubated with rabbit anti-RAGE antibody (1:2,000, Sigma), anti-NLRP3 polyclonal antibody (1:1,000 NBP2-12446 Novus Biological), anti-IL-1β (1:1,000 sc-7884 Santa Cruz), anti-IL-18 (1:1,000 sc-7954 Santa Cruz), rabbit anti-NF-KB (1:2,000 Cell Signal), and anti-α-tubulin antibody (1:1,000, Cell Signal) overnight at 4°C, and then with a horseradish peroxidase-conjugated secondary goat anti-rabbit IgG (1:2,000, Santa Cruz) for 2 hours at room temperature. SuperSignal West Pico Chemiluminescent Substrate (Thermo Scientific, Rockford, USA) was used for protein detection. Bands were visualized by a Super Western Sensitivity Chemiluminescence Detection System (proteinsimple, CA, USA). Autoradiographs were quantified by densitometry (NIH Image J). Each blot was representative of at least three comparable independent experiments.

### 4. Histopathological analysis

Mice were humanely killed after being anesthetized. Eyeball exsanguination, followed by cardiac exsanguination, were performed post sacrifice. Kidneys were dissected and weighed (wet weight) before snap frozen in liquid nitrogen, and were stored at -80°C or in 4% paraformaldehyde before being embedded in paraffin. From each sample, 4-μm sections were obtained and then stained with periodic acid–Schiff (PAS) for measurement of mesangial areas, which were calculated as the percentage of glomerular area from digital pictures of glomeruli using Image J 1.49V software. An average of 15–20 glomeruli per kidney per animal was examined. Sections were also stained with anti-NLRP3 polyclonal antibody (1:250 NBP2-12446 rabbit anti-NLRP3, Novus Biological, UK), anti-NF-*κ*B (1:250 4764 rabbit anti-NF-KB p65, Cell Signaling Technology, US), anti-IL-1β (1:250 sc-7884 rabbit polyclonal anti-IL-1β, Santa Cruz, US), anti-IL-18 (1:250 sc-7954 rabbit polyclonal anti-IL-18, Santa Cruz, US), and anti-RAGE antibody (1:250 SAB2105049 rabbit anti-receptor for Advanced glycation end products, Sigma-Aldrich, US). For glomerular assessment, 15 glomeruli were examined in photomicrographs (Nikon eclipse Ti-U, Japan) under an identical light condition, and the percentage of areas of the glomerular tuft stained was digitally quantitated based on red, green, and blue hues (Image Pro-Plus 6.0 software, Media Cyber Netics, Silver Spring, MD). An average of 6 fields (magnification, 3200x) was assessed for each animal (n = 6~8 per group).

### 5. Statistical analysis

Results are expressed as mean ± standard error of mean (SEM). Analyses were carried out using the SPSS software version 16.0 for Windows (SPSS Inc., Chicago, US). Our primary statistical test was one-way analysis of variance (ANOVA). If ANOVA results were significant, a post hoc comparison between groups was performed using the Student-Newman-Keuls test. A *P* value less than 0.05 was considered statistically significant.

## Results

### 1. Baseline characteristics and metabolic parameters in PIO treated apoE (-/-) and diabetic apoE (-/-) mice

As shown in Table 1, PIO treatment significantly lowered blood glucose, serum BUN, and creatinine in diabetic apoE (-/-) mice (*P* < 0.05 *vs*. no treatment group), but these effect were not observed among non-diabetes groups. Treatment of apoE (-/-) and diabetic apoE (-/-) groups with PIO for 8 weeks resulted in a decrease in serum cholesterol compared to those without treatment (*P* < 0.05 *vs*. no treatment group), but there were no differences in serum triglycerides. PIO not only improved blood glucose and cholesterol levels, but also partly reversed the decline in the estimated glomerular filtration rate (eGFR). Compared to the control, apoE (-/-) and diabetic apoE (-/-) mice had higher kidney weight (KW), body weight (BW), and ratio of KW to BW (*P* < 0.05 *vs*. control group), while PIO treatment had no effect on KW and BW in each group.

**Table 1:** Baseline characteristics and metabolic parameters in PIO treated apoE (-/-) and diabetic apoE (-/-) mice. Values represent means ± standard error (SE) for blood glucose, serum cholesterol, BUN, creatinine, triglyceride, kidney weight, body weight, and KW/BW; n = 6–8 per group for the 5 groups: apoE (-/-) mice and diabetic apoE (-/-) mice with and without pioglitazone treatment (100 mg/kg/day), and control. *P < 0.05 *vs*. apoE (-/-) group. ^#^*P* < 0.05 *vs*. diabetic apoE (-/-) group. ^&^*P* < 0.05 *vs*. apoE (-/-) and diabetic apoE (-/-) group.

	ApoE (-/-)		ApoE (-/-) + diabetes		Control
PIO	-	+	-	+	_
Blood glucose (mmol/L)	9.70±1.14	8.36±1.81	15.41±4.88	8.86±1.23^#^	6.78±2.62
Cholesterol (mmol/L)	12.66±1.41	9.84±0.84*	16.07±2.02	12.53±1.06^#^	2.04±0.26
Kidney weight (mg)	398.26±84.29	326.82±59.94	359.80±63.17	331.46±24.18	250.92±47.93^&^
Body weight (g)	33.57±2.79	30.89±3.50	33.20±2.33	31.68±2.03	26.21±4.43^&^
BUN (mmol/L)	13.42±4.21	7.56±1.55*	18.58±7.81	8.81±1.51^#^	7.02±1.98
Creatinine (μmol/L)	18.20in.42	9.96±1.55*	36.20±13.4	13.20±2.1^#^	10.40±2.07
Triglyceride (mmol/L)	1.05±0.45	0.96±0.18	1.19±0.49	1.10±0.17	0.73±0.16
KW/BW	11.80±1.90	10.57±1.46	10.90±2.14	10.48±0.66	13.95±1.21^&^

### 2. The effect of PIO on expression of renal AGE/RAGE and NF-*κ*B

**Fig 1:**
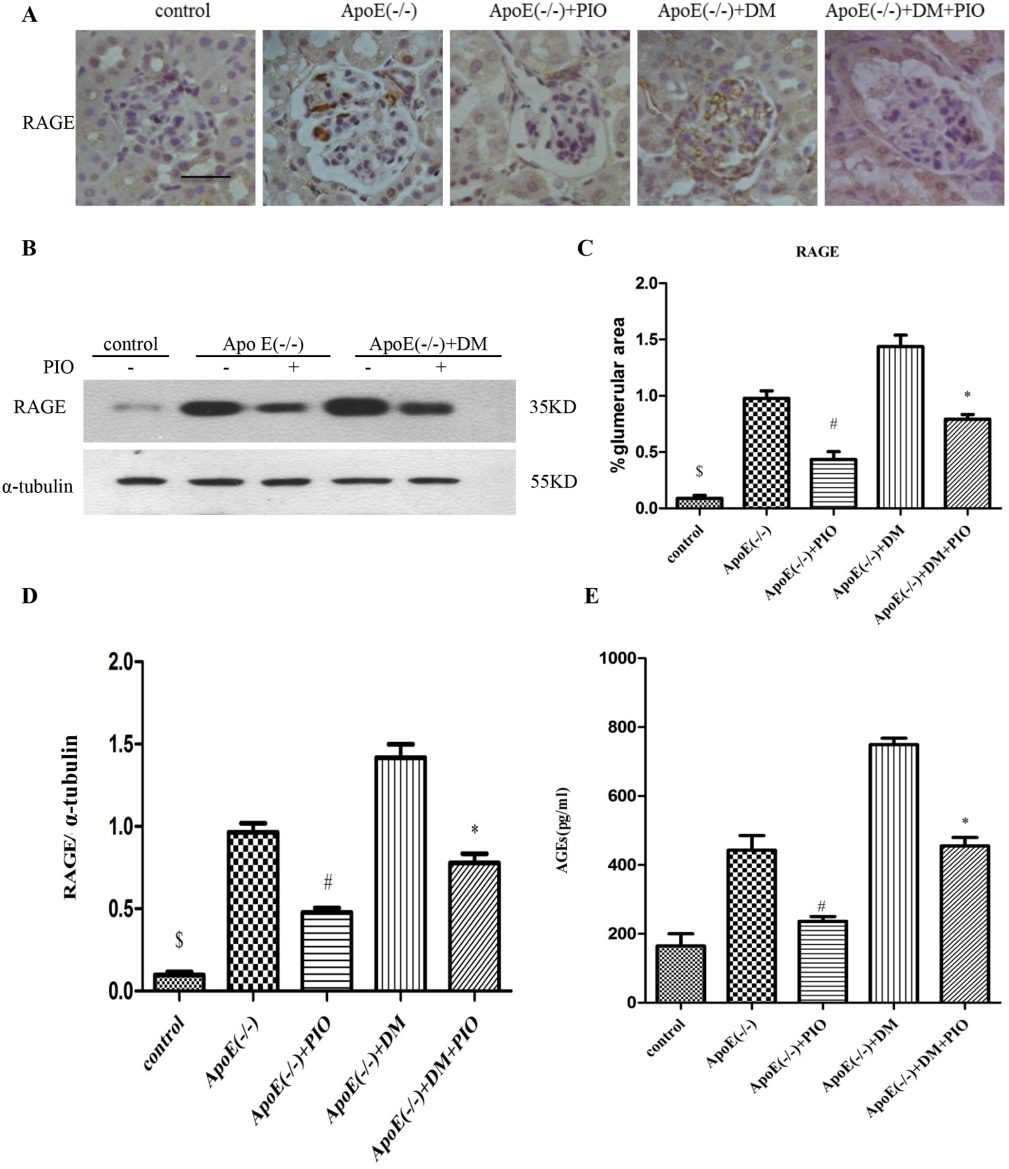
PIO inhibits expression of the renal AGE/RAGE axis in diabetic apoE (-/-) mice. (A) Immunostaining for RAGE in control, apoE (-/-); apoE (-/-) + pioglitazone 100 mg/kg/day, diabetic apoE (-/-); diabetic apoE (-/-) + pioglitazone 100 mg/kg/day. Scale bar in control = 50 μm. (B) The renal cortex RAGE detected by Western blotting in control, apoE (-/-); apoE (-/-) + pioglitazone 100 mg/kg/day, diabetic apoE (-/-); diabetic apoE (-/-) + pioglitazone 100 mg/kg/day. (C) Digital quantification of glomerular staining for RAGE for n = 6–10 per group. (D) The relative RAGE levels were determined after normalization with α-tubulin (n = 3). (E) The renal cortex AGEs detected by ELLSA in control; apoE (-/-); apoE (-/-) + pioglitazone 100 mg/kg/day, diabetic apoE (-/-); diabetic apoE (-/-) + pioglitazone 100 mg/kg/day and control (n = 3). ^#^*P* < 0.05 *vs*. apoE (-/-), *P < 0.05 *vs*. diabetic apoE (-/-), ^&^*P* < 0.05 *vs*. apoE (-/-) and diabetic apoE (-/-) group.

**Fig 2:**
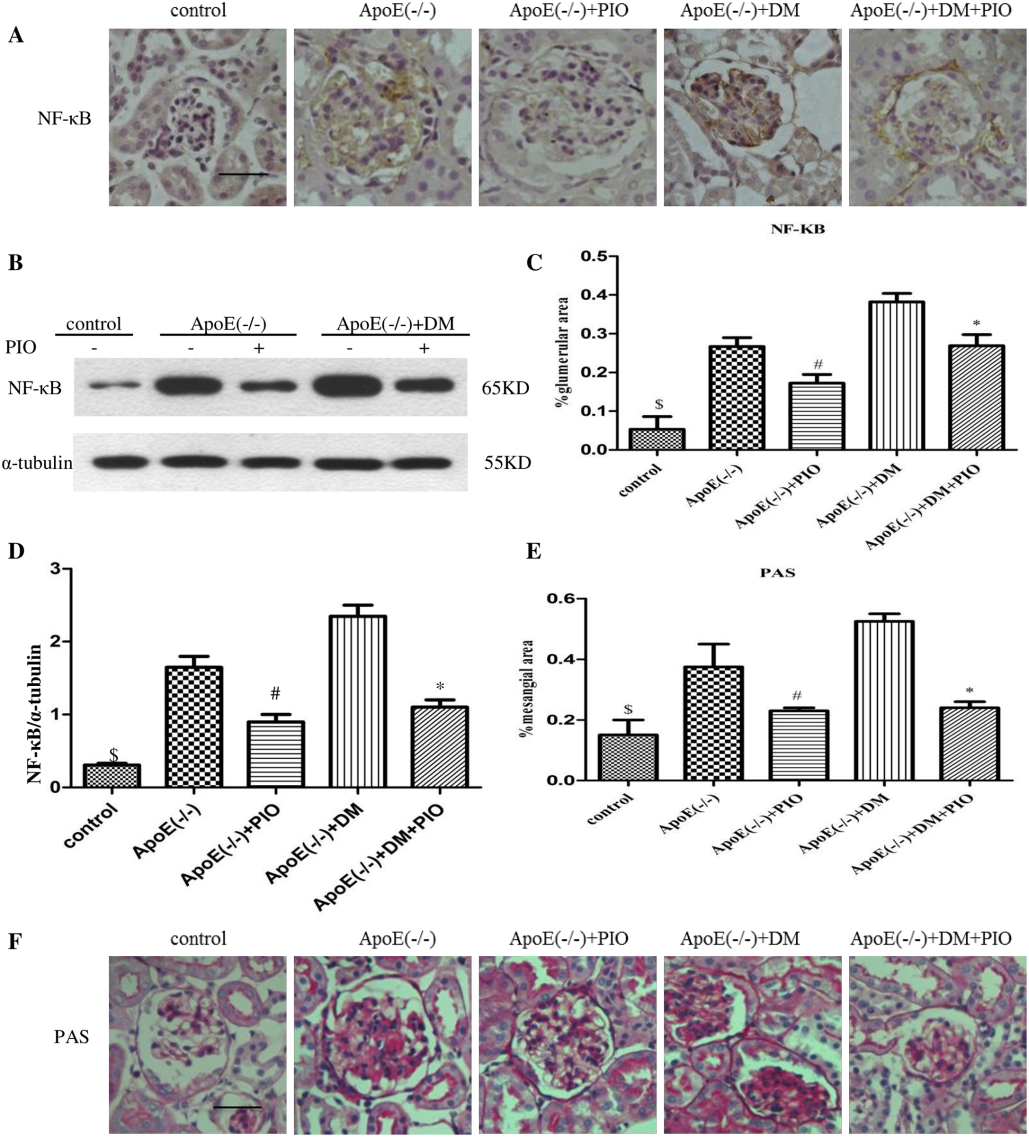
PIO inhibits expression of renal NF-κB and mesangial expansion in diabetic apoE (-/-) mice. (A) Immunostaining for NF-κB in control, apoE (-/-); apo E (-/-) + pioglitazone 100 mg/kg/day, diabetic apoE (-/-); diabetic apoE (-/-) + pioglitazone 100 mg/kg/day. Scale bar in control = 50 μm. (B) The renal cortex NF-κB detected by Western blotting in control, apoE (-/-); apoE (-/-) + pioglitazone 100 mg/kg/day, diabetic apoE (-/-); diabetic apoE (-/-) + pioglitazone 100 mg/kg/day. (C) Digital quantification of glomerular staining for NF-κB for n = 6–10 per group. (D) The relative NF-κB levels were determined after normalization with α-tubulin (n = 3). (F) PAS staining in control; apoE (-/-); apoE (-/-) + pioglitazone 100 mg/kg/day, diabetic apoE (-/-); diabetic apoE (-/-) + pioglitazone 100 mg/kg/day. Scale bar in control = 50 μm. (E) Digital quantification of mesangial area for n = 6–8 per group. ^#^*P* < 0.05 *vs*. apoE (-/-), *P < 0.05 *vs*. diabetic apoE (-/-), ^&^*P* < 0.05 *vs*. apoE (-/-) and diabetic apoE (-/-) group.

Diabetes was associated with increased expression of AGE and RAGE in cortical lysates. The changes in the AGE/RAGE axis were significant in both diabetic apoE (-/-) and apoE (-/-) mice, while diabetic apoE (-/-) mice exhibited more prominent changes compared to apoE (-/-) mice. PIO treatment reduced the expression of molecules in the AGE/RAGE axis (Fig 1). Glomerular expression of RAGE and NF-*κ*B also increased in diabetic apoE (-/-) and apoE (-/-) mice, with more prominent changes in diabetic apoE (-/-) mice. PIO treatment reduced the glomerular expression of RAGE and NF-*κ*B (Fig 2). After 20 weeks of diabetes, the mesangial areas significantly increased in diabetic apoE (-/-) mice compared to apoE (-/-) mice, and the mesangial expansion was significantly attenuated by PIO treatment (Fig 2).

### 3. The effect of PIO on the NLRP3 inflammasome and expression of its downstream inflammatory molecules

**Fig 3:**
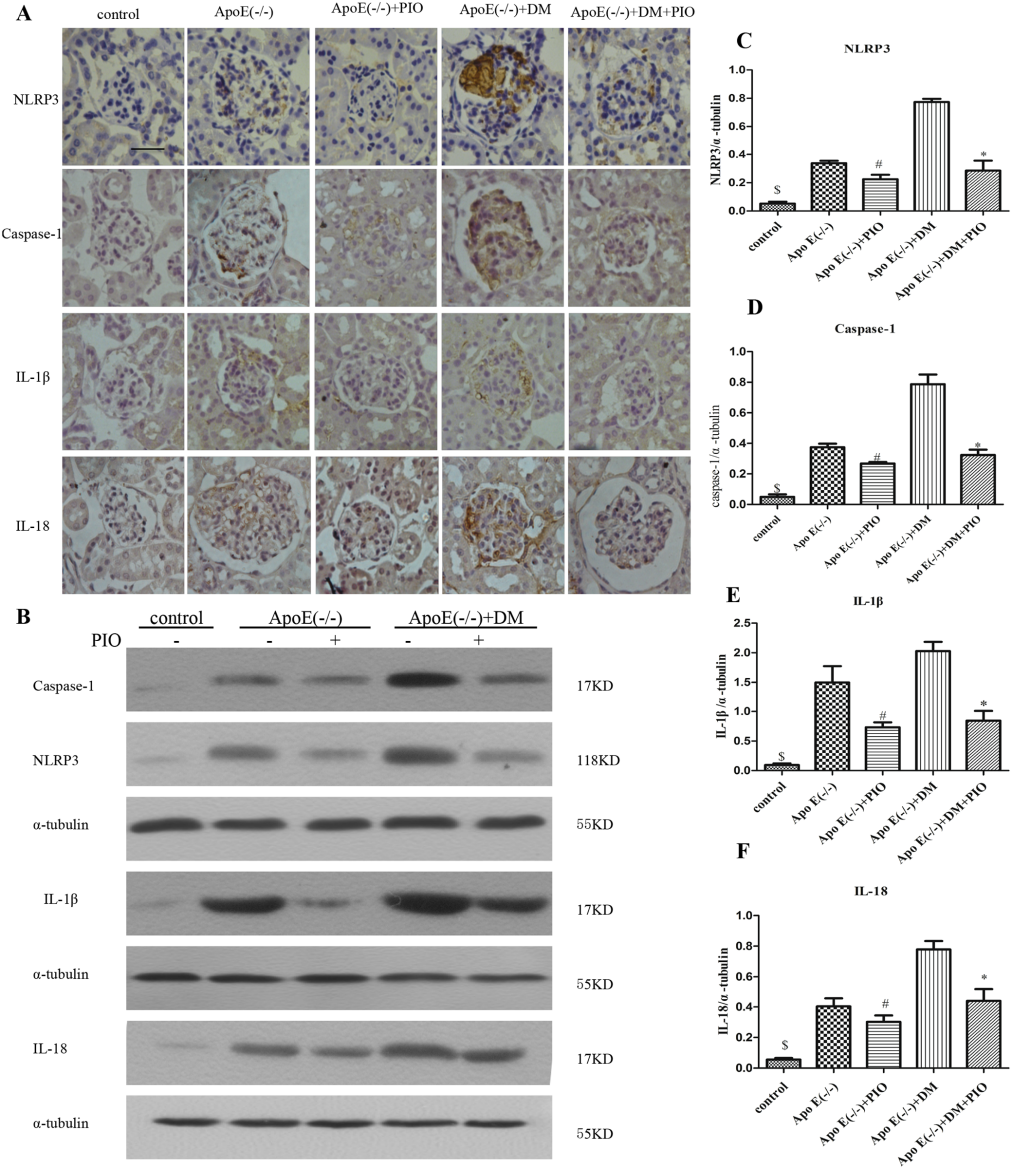
PIO inhibits expression of the renal NLRP3 inflammasome in diabetic apoE (-/-) mice. (A) Immunostaining for NLRP3, caspase-1, IL-18, IL-1β in control; apo E (-/-); apo E (-/-) + pioglitazone 100 mg/kg/day, diabetic apoE (-/-); diabetic apoE (-/-) + pioglitazone 100mg/kg/day. Scale bar in control = 50 μm for n = 6–10 per group. (B-F) Quantification of NLRP3, caspase-1, IL-18. And IL-1β protein levels in the renal cortex as determined by Western blot analysis in control, apoE (-/-), apoE (-/-) + pioglitazone 100 mg/kg/day, diabetic apoE (-/-), diabetic apoE (-/-) + pioglitazone 100 mg/kg/day groups. Relative protein levels were determined after normalization with α-tubulin (n = 3). ^#^*P* < 0.05 *vs*. apoE (-/-), *P < 0.05 *vs*. diabetic apoE (-/-), ^&^*P* < 0.05 *vs*. apo E (-/-) and diabetic apo E (-/-) group.

NLRP3, caspase-1, IL-1β, and IL-18 were minimally expressed in normal glomerular tissues, but were significantly up-regulated in diabetic apoE (-/-) and apoE (-/-) mice, with more prominent changes in diabetes apoE (-/-) mice. PIO treatment reduced expression of these molecules compared to those in the treatment groups (*P* < 0.05; Fig 3)

## Discussion

In the current study, we found that 8-week treatment with PIO significantly ameliorated the diabetes-induced increases in serum glucose, cholesterol, BUN, and creatinine in apoE (-/-) mice. However, PIO exhibited no effect on the diabetes-induced increases in absolute KW and KW/BW ratios. In PIO-treated apoE (-/-) and diabetic apoE (-/-) mice, activation of NF-*κ*B and the AGE/RAGE axis were partially suppressed. PIO significantly attenuated the diabetes-induced activation of the NLRP3 inflammasomes and their downstream effectors, including caspase-1, IL-1β, and IL-18. In light of these findings, PIO could delay the progression of DN in mice with type 1 diabetes mellitus.

DN is a sterile inflammatory disease. The accumulation of inflammatory cells in the kidney is observed during the progression of DN, and the severity of inflammation is closely associated with the deterioration of renal function [13,14]. In a series of experiments using *in vitr*o models [15], intrarenal inflammasome activation elevated intrarenal IL-1β and NLRP3 mRNA levels were increased in both db/db mice (modelling type 2 diabetes) and murine streptozotocin-induced type 1 diabetic mice. Two mechanisms are required for NLRP3 activation. The first one is driven by Toll-like receptors (TLR), tumor necrosis factor receptor (TNFR), or IL-1R signaling, which is required for the activation of NF-*κ*B at the transcriptional level. This process is very active during diabetes, leading to IL-1 and IL-18 gene expression [16,17]. The other mechanism is the enzymatic cleavage pro-IL-1b and pro-IL-18, which is required for the secretion of active cytokines into the extracellular space. This process requires the oligomerization of NLRP3 with adapter proteins, such as ASC, which facilitates the proteolytic cleavage of pro-caspase-1 to the active form, caspase-1. Caspase-1 then cleaves the pro-cytokines, generating mature IL-1β and IL-18 that can be secreted. These processes are driven by various activators, including ROS.

The accumulation of AGEs during diabetes contributes to the development and the progression of renal damage [18]. Both RAGE-dependent and the AGE/RAGE axis play a pivotal role in DN progression, and inhibiting AGEs accumulation is found to be reno-protective in experimental models of diabetes [19]. Suppressing the AGE/RAGE axis can effectively attenuate the development of diabetes-associated glomerular fibrosis, although RAGE-independent signaling can also be activated *in vivo* in diabetic kidneys by AGEs [20]. Targeting the AGE/RAGE axis might have synergistic effects for the prevention of DN [20]. AGEs are known to induce mitochondrial ROS generation, a promoter of DN [21], by binding to RAGE [22], and the inhibition of the AGE/RAGE axis has been proven to decrease tissue ROS levels [23]. Furthermore, treatment of diabetic mice with mitotempo *in vivo* or p66shc deficiency inhibits renal NLRP3 expression and IL-1β cleavage, as well as reduces the extent of albuminuria and mesangial expansion. Consistent with these studies, we found that hyperglycemia and **hyperlipidemia** significantly increase renal cortical and circulating AGE levels in diabetic apoE (-/-) mice. Activation of the AGE/RAGE axis was accompanied by activation of the NLRP3 inflammasome, leading to increased ROS production.

NF-*κ*B is necessary for macrophage production of IL-1β, and can activate IL-1β signaling through inducing IL-1R and Interluekin1 receptor-associated kinase-4 (IRAK4). NF-*κ*B participates in regulating the expression of pro-inflammatory cytokines, chemokines, and adhesion molecules, and contributes to macrophage infiltration in rodent models of DN [24]. NF-*κ*B can activate NLRP3 signaling in macrophages as well. Furthermore, the NF-*κ*B inhibitor has been found to protect against organ damage in db/db mice through effects that go beyond those on glucose homeostasis, including the inhibition of pro-fibrotic and pro-inflammatory processes [25]. Both NF-*κ*B and the AGE/RAGE axis have been proven to activate the glomerular NLRP3 inflammasome [26]. Consistent with these studies, we found a significant increase in the activation of NF-*κ*B and AGE/RAGE axis in the kidneys of apoE (-/-) and diabetic apoE (-/-) mice, as well as the activation of glomerular NLRP3 inflammasomes.

During hyperglycemia and hypercholesterolemia, NLRP3 initiates or amplifies diverse downstream signaling pathways and drives pro-inflammatory processes [27], leading to cellular damage, such as autophagy and pyroptosis [28]. In residential cells of the kidneys, such as podocytes, endothelial cells, or mesangial cells [3], inflammatory cells (but not bone marrow–derived cells) can secrete NLRP3 inflammasome-associated cytokines, and thus aggravate DN [29]. Indeed, kidney-limited silencing of ASC can attenuate proteinuria, albuminuria, and glomerular sclerosis [30]. NLRP3 is the critical component of the inflammasomes and plays an independent role in injury signaling, apart from that of other inflammasome components such as ASC and caspase-1 [31]. Studies have shown that, in patients with type 2 diabetes mellitus, NLRP3 inflammasomes were up-regulated, and metformin treatment modulated the activation of these inflammasomes [32]. However, whether inflammasomes are activated in the glomeruli during DN is still unclear. In our study, we found that in normal glomeruli, NLRP3 inflammasome and pro-cytokine expression is nearly absent, while apoE (-/-) and diabetic apoE (-/-) mice had an obvious activation of NLRP3 and its downstream effectors, including caspase-1, IL-1β, and IL-18. The results were demonstrated by immunostaining and Western blot analyses. The pathologic changes were accompanied by higher serum BUN, creatinine, and glomerular mesangial expansion.

Long-term use of PPAR-γ activators has been shown to improve glucose homeostasis by restoring insulin sensitivity. Although PPAR-γ is predominantly expressed in adipocytes, it is also found in vascular tissues, inflammatory cells, and renal glomerular, as well as tubular cells [33], all of which are involved in DN pathogenesis. The PPAR-γ agonist thiazolidinedione (TZD) can improve insulin sensitivity and has great therapeutic potential for controlling type 2 diabetes mellitus [34]. Preclinical studies showed that PPAR-γ agonists also reduced microalbuminuria and prevented glomerular hyper-filtration and extracellular matrix protein accumulation in streptozotocin-induced diabetic rats, independent of their insulin-sensitization [35]. Furthermore, studies using rodent models of DN and non-diabetic nephropathy also showed that the reno-protective effect of TZDs might go beyond their effects on blood pressure and glucose homeostasis [36]. TZDs can limit inflammatory responses, judging from the observation that rosiglitazone, another PPAR-γ agonist, significantly attenuated stretch-induced NF-κB activation, the production of monocyte chemoattractant protein-1 (MCP-1), and monocyte chemotaxis [37]. Therefore, it has been suggested that PPAR-γ agonists may show promise as therapeutic agents for DN. PPAR-γ agonists have also been shown to affect the activation of inflammasomes, as there are reports suggesting that PPAR-γ agonists prevented nucleotide-binding domain, leucine-rich repeat/pyrin domain-containing-3 (NALP3) inflammasome formation, and IL-1β production in HK-2 cells stimulated by monosodium urate crystals [38]. Evidence also indicates that PPAR-γ mitigates inflammation, as PPAR-γ activation suppresses nuclear factor of activated T cells (NFAT) signaling and reduces expression of NADPH oxidase subunits, resulting in lower levels of ROS production [13,39]. PPAR-γ also down-regulates TLR2 and TLR4 signaling by either blocking TLR expression or suppressing the downstream NF-*κ*B and AP-1-dependent pathways [40]. The effects of PPAR-γ on NF-*κ*B seem to be mediated by a combination of direct physical interactions, sequestration of NF-*κ*B co-activators, and transcriptional control of NF-*κ*B-related pro-inflammatory genes [41]. In our study, we discovered that in diabetic apoE (-/-) mice, the NLRP3 inflammasomes are significantly higher compared to levels in the other groups. PIO not only assisted in controlling blood glucose and lipids and reducing NF-*κ*B and AGE/RAGE levels in the glomeruli, but PIO also suppressed the activation of the glomerular NLRP3 inflammasomes, as well as their downstream molecules including caspase-1, IL-1β, and IL-18, which has not been reported before. As our results indicated, PIO treatment can ameliorate activation of NLRP3 inflammasomes, caspase-1, IL-1β, and IL-18 in apoE (-/-) mice; this means that the beneficial effect of PIO is independent of its hypoglycemic effect. The inhibition of inflammasome in patients with diabetes may be an important therapeutic approach.

## Conclusion

In conclusion, hyperglycemia and hyperlipidemia can lead to the activation of NLRP3 inflammasomes, and their downstream molecules, including caspase-1, IL-1β, and IL-18, in the glomerular mesangium. PIO treatment can inhibit the secretion of inflammatory cytokines through mechanisms including suppression of the AGE/RAGE signaling pathway and local ROS release, as well as lowering NF-*κ*B levels, both of which attenuate NLRP3 infalmmasome activation and reduce diabetic renal injury. Our findings show that PIO, a PPAR-γ agonist, ameliorate DN and this is associated with downregulation of the NLRP3 inflammasome, potentially providing a new underlying mechanism responsible for the beneficial effect of PIO for DN.
